# Genetic and Phenotypic Characteristics of Congenital Hypothyroidism in a Chinese Cohort

**DOI:** 10.3389/fendo.2021.705773

**Published:** 2021-09-03

**Authors:** Wei Long, Fang Guo, Ruen Yao, Ying Wang, Huaiyan Wang, Bin Yu, Peng Xue

**Affiliations:** ^1^Department of Medical Genetics, Affiliated Changzhou Maternal and Child Health Care Hospital, Nanjing Medical University, Changzhou, China; ^2^Department of Medical Genetics and Molecular Diagnostic Laboratory, Shanghai Children’s Medical Center, Shanghai Jiao Tong University School of Medicine, Shanghai, China; ^3^Department of Pediatrics, Affiliated Changzhou Maternal and Child Health Care Hospital, Nanjing Medical University, Changzhou, China; ^4^Department of Pediatrics, Affiliated Changzhou Children’s Hospital of Nantong University, Changzhou, China

**Keywords:** hypothyroidism, whole-exome sequencing, biallelic variant, oligogenic variant, characteristic

## Abstract

**Background:**

The molecular etiology and the genotype–phenotype correlation of congenital hypothyroidism (CH) remain unclear.

**Methods:**

We performed genetic analysis in 42 newborns with CH using whole-exome sequencing. Patients were divided into a single-gene group and a multi-gene group according to the number of affected genes, or divided into a monoallelic group, a biallelic group, and an oligogenic group according to the pattern of the detected variants. The clinical characteristics were compared between groups.

**Results:**

Thyroid dysgenesis (TD) was observed in 10 patients and goiter in 5 patients, whereas 27 patients had normal-sized gland-in-situ (GIS). We identified 58 variants in five genes in 29 patients. The genes with the most frequent variants were *DUOX2* (70.7%), followed by *TSHR* (12.1%), *DUOXA2* (10.3%), and *TPO* (5.2%). Variants in the genes causing dyshormonogenesis (DH) were more common than those in the genes causing TD (87.9% *versus* 12.1%). Among the patients with detected variants, 26 (89.7%) were harboring a single gene variant (single-gene group), which include 22 patients harboring biallelic variants (biallelic group) and four patients harboring monoallelic variants (monoallelic group). Three (10.3%) patients harbored variants in two or three genes (multi-gene group or oligogenic group). Compared with the single-gene group, the levothyroxine (L-T4) dose at 1 year of age was higher in the multi-gene group (*p* = 0.018). A controllable reduction in the L-T4 dose was observed in 25% of patients in the monoallelic group and 59.1% of patients in the biallelic group; however, no patients with such reduction in the L-T4 dose were observed in the oligogenic group.

**Conclusions:**

Patients with normal-sized GIS accounted for the majority of our cohort. Genetic defects in the genes causing DH were more common than those in the genes causing TD, with biallelic variants in *DUOX2* being dominant. DH might be the leading pathophysiology of CH in Chinese individuals.

## Introduction

Congenital hypothyroidism (CH) is the most common neonatal endocrine disorder with an incidence in newborns from approximately 1/2,000 to 1/4,000 (1). Delayed treatment of CH might result in profound neurodevelopmental delay. A newborn screening (NBS) program has been implemented to facilitate the prompt diagnosis of CH, and levothyroxine (L-T4) has also been used in the treatment of CH. However, the etiology of CH remains unclear.

Numerous studies have identified genetic defects in patients with CH (2). According to the pathophysiology, CH is traditionally subdivided into thyroid dysgenesis (TD) and dyshormonogenesis (DH). TD, which is caused by abnormal development of the thyroid gland, has been reported to be associated with mutations in the following genes: thyroid-stimulating hormone receptor (*TSHR*), NK2 homeobox 1 (*NKX2-1*), NK2 homeobox 5 (*NKX2-5*), forkhead box E1 (*FOXE*1), paired box 8 (*PAX8*), and GLIS family zinc finger 3 (*GLIS3*) (3). DH has been associated with mutations in genes encoding known components of the thyroid hormone biosynthesis machinery (4), such as dual oxidase 2 (*DUOX2*), dual-oxidase maturation factor 2 (*DUOXA2*), thyroglobulin (*TG*), thyroid peroxidase (*TPO*), solute carrier family 5 member 5 (*SLC5A5*), solute carrier family 26 member 4 (*SLC26A4*), and iodotyrosine deiodinase (*IYD*) (5, 6).

To date, more than 20 genes have been reported to be involved in the pathogenesis of primary CH (2), and new genetic defects continue to be identified due to the use of efficient genetic approaches, such as target region sequencing and whole-exome sequencing (WES) (6). However, these reported genes cannot fully explain the molecular etiology of CH. In the present study, we performed screening of variants using WES and collected clinical data in a cohort of Chinese patients with CH in order to analyze the relationship between the genotype and the phenotype in CH. This is a comprehensive study that evaluates genotypic and phenotypic correlations through implementation of WES detecting genetic defects in a cohort.

## Methods

### Patients

Changzhou Maternal and Child Health Care Hospital is the only NBS institution in Changzhou. Participants were patients who were diagnosed and treated at this hospital. They all volunteered to participate in this study after informed consent. CH was diagnosed based on findings of elevated levels of serum thyroid-stimulating hormone (TSH; ≥9 mIU/L) and low levels of free thyroxine (FT4; <7.77 pmol/L) for newborns who were positive in the NBS program (heel blood TSH, ≥9.0 mIU/L). The levels of serum TSH and FT4 were determined by electrochemistry immunoassay using the COBAS e601 analyzer (Roche Diagnostics, Mannheim, Germany). The levels of heel blood TSH were detected by time-resolved fluoroimmunoassay using the Wallac 1235 AutoDELFIA (Perkin Elmer, Waltham, MA, USA). Following confirmation of diagnosis, the patients were immediately administered L-T4 at an initial dose of 10–15 μg kg^−1^ day^−1^. Patients with other congenital diseases or those whose mothers were diagnosed with Graves’ disease were excluded from the study. The study design and protocol were approved by the Ethics Committee of the Nanjing Medical University (approval no. 2019-258). Written informed consent for participation in this study was provided by the participants’ legal guardians.

### Collection of Clinical Data and Blood Samples

All newborns with CH were treated and followed up at the Department of Medical Genetics of Changzhou Maternal and Child Health Care Hospital. The levels of heel blood TSH of newborns at screening and those of serum TSH and FT4 at diagnosis were recorded in the electronic NBS information system. All participants were followed up until the conclusion of the present study. The administered dose of L-T4 during treatment was managed and recorded by a clinician. Patients with controllable reduction of L-T4 dose were defined as patients whose levels of thyroid hormones were normal within 2 months of treatment and whose L-T4 doses were reduced or equal compare to the previous follow-up. The reference ranges for the treatment of CH are shown in **Table 1**. Thyroid morphology was determined using ultrasound scanning. The methods and reference values of thyroid volumes were based on a published study on Chinese newborns (7). Venous blood was sampled from the proband and the proband’s parents and then stored in an ultra-low-temperature refrigerator.

**Table 1:** Reference ranges in the treatment of patients with congenital hypothyroidism (CH).

Age	TSH (mIU/L)	FT4 (pmol/L)
2–20 weeks	1.7–9.1	11.6–29.6
5–24 months	0.8–8.2	10.3–23.2
2–7 years	0.7–5.7	12.8–27

TSH, thyroid-stimulating hormone; FT4, free thyroxine.

### Whole-Exome Sequencing

WES and variant analysis were performed in the Department of Medical Genetics and Molecular Diagnostic Laboratory of Shanghai Children’s Medical Center. Briefly, genomic DNA was extracted from blood samples of patients and their parents using the Gentra Puregene Blood Kit (Qiagen, Hilden, Germany) according to the manufacturer’s protocol. Whole-exome capture was performed using an Agilent SureSelect V6 enrichment capture kit (Agilent Technologies, Inc., Woburn, MA, USA) according to the manufacturer’s instructions. The captured library was sequenced using an Illumina HiSeq 2500 System (Illumina, Inc., San Diego, CA, USA). Original sequencing data were assessed using FastQC (version 0.11.2) for quality control. The Burrows–Wheeler Alignment (BWA) tool (v.0.2.10) was employed for sequencing data alignment to the Genome Reference Consortium Human Build 37 (GRCh37/hg19). Single nucleotide variants and small indels were identified using the Genome Analysis Toolkit (GATK). All variants were saved in VCF format and uploaded to the Ingenuity Variant Analysis (Ingenuity Systems, Redwood City, CA, USA) and the TGex (Translational Genomics Expert) platform for biological analysis and interpretation. Variants were classified based on the guidelines of the American College of Medical Genetics and Genomics (ACMG) (8), excluding benign and likely benign, but including *DUOX2* p.His678Arg, which has been reported as a functional SNP (9). Variants detected by WES were confirmed by Sanger sequencing in each patient and the parents.

### Patient Classification and Comparison of Characteristics

Patients were assigned into groups using two different strategies: 1) based on the number of affected gene—the single-gene group comprised cases with variants in a single gene, while the multi-gene group comprised cases with variants in multiple genes; 2) based on the pattern of the detected variants—the monoallelic group comprised cases with a single variant in a single gene, the biallelic group comprised cases with homozygous variants or compound heterozygous variants in a single gene determined by analysis of the parents, and the oligogenic group included cases with two or more variants in different genes. The clinical characteristics, such as heel blood TSH at NBS, serum TSH and FT4 at diagnosis, the initial L-T4 dose, the L-T4 dose at 1 year of age, current L-T4 dose, and thyroid morphology, were analyzed among the groups.

### Statistical Analysis

The summary statistics for non-normally distributed quantitative variables were expressed as the median plus interquartile range (IQR). Categorical data were summarized as number and percentages. The Mann–Whitney *U* test or the Kruskal–Wallis test was performed for comparisons between two or more groups, and Dunn’s test was used for comparisons between subgroups. The chi-square test was used for categorical variables, whereas Fisher’s exact test was used if the expected cell count was less than five. Differences were considered statistically significant at a two-sided *p*-value of 0.05. All statistical tests were performed using R version 3.6.3.

## Results

### Variant Frequencies in Participants

Following acquisition of consent forms from guardians, 42 newborns with CH (21 males and 21 females) were enrolled in our study, including 40 non-consanguineous individuals and two siblings. The birth years of these participants ranged from 2011 to 2019. During this period, there were a total of 295,650 newborns, and 133 of them were diagnosed with CH. The average birth weight of the enrolled newborns was 3,346 g (range from 2,350 to 4,350 g), while the average gestational age was 39 weeks (from 34 + 6 to 42 weeks). We observed the occurrence of TD in 10 patients and goiter in five patients, whereas 27 patients had normal-sized gland-in-situ (GIS).

**Figure 1:**
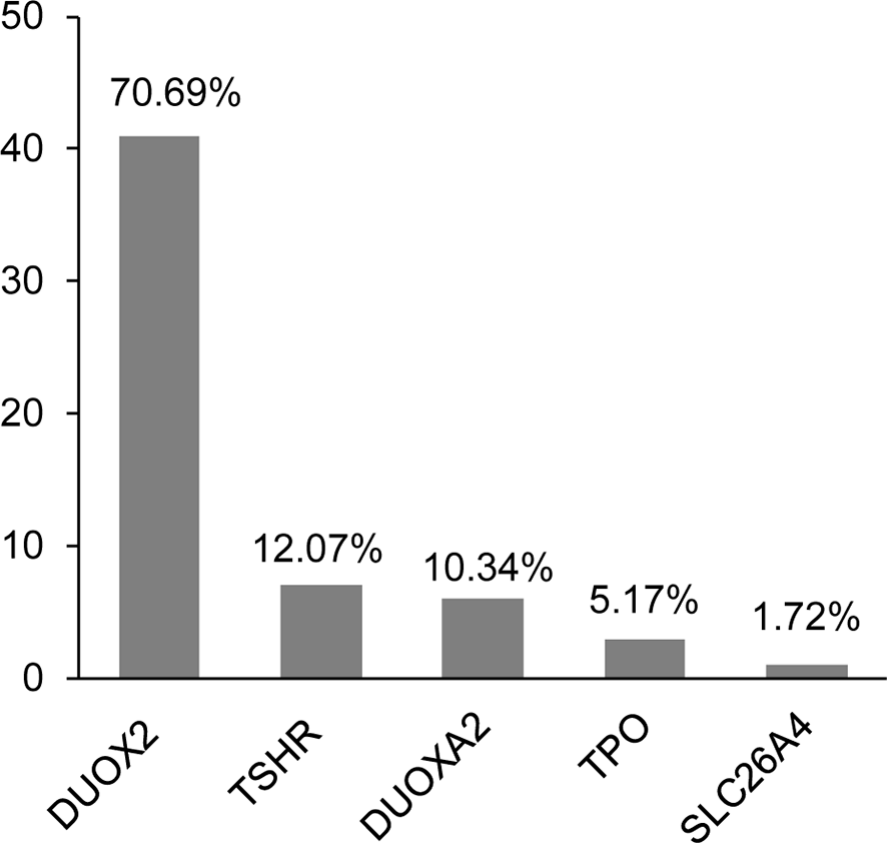
Distribution of the 58 detected variants in five genes.

In total, we identified 58 variants in five genes—namely, *DUOX2*, *TSHR*, *DUOXA2*, *TPO*, and *SLC26A4*—in 29 of the participants (29/42, 69.0%). The positive rate of the variants was 75.0% (24/32) in patients with DH, whereas it was 50% (5/10) in patients with TD. The clinical characteristics and all variants are shown in **Supplementary Table S1**. Most of the identified variants were heterozygous, except for two homozygous *DUOX2* mutations (i.e., p.Lys530* and p.Arg1110Gln) detected in two patients. Most variants were included in the databases or reported in previous studies, except for one heterozygous variant in *TSHR* (i.e., p. Ala579Val) that was novel. Interestingly, we found that variants in the genes causing DH were more common than those in the genes causing TD (87.9% *versus* 12.1%). We further observed that, among the 58 identified variants, the genes with the most frequent variants were *DUOX2* (70.7%), followed by *TSHR* (12.1%), *DUOXA2* (10.3%), and *TPO* (5.2%) (**Figure 1**). In addition, seven of these variants were detected in more than one patient: five *DUOX2* variants, one *DUOXA2* variant, and one *TSHR* variant (**Table 2**). We found that these variants accounted for 53.4% (31/58) of the total variants, with the p.Lys530* and p.Arg1110Gln mutations in *DUOX2* constituting the predominant sites in the present cohort.

**Table 2:** Variants detected in multiple participants.

Gene	Nucleotide change	Amino acid change	No. of cases	Detection rate in the present cohort (%)
*DUOX2*	c.1588A>T	p.Lys530*	8	19.0
*DUOX2*	c.3329G>A	p.Arg1110Gln	8	19.0
*DUOX2*	c.2654G>A	p.Arg885Gln	3	7.1
*DUOXA2*	c.413dupA	p.Tyr138*	3	7.1
*TSHR*	c.1574T>C	p.Phe525Ser	3	7.1
*DUOX2*	c.2033A>G	p.His678Arg	2	4.8
*DUOX2*	c.2048G>T	p.Arg683Leu	2	4.8

*Stop-gain variant.

### Characteristics in the Single-Gene and Multi-Gene Groups

**Figure 2:**
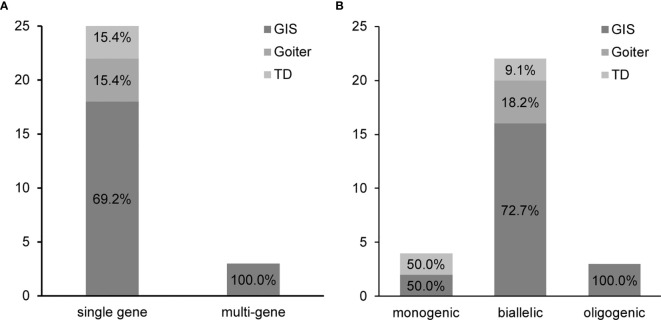
Grouping of 29 patients with congenital hypothyroidism (CH) causative variants. **(A)** Patients were divided into a single-gene and a multi-gene group. **(B)** Patients were classified into a monoallelic, a biallelic, and an oligogenic group. *GIS*, gland*-*in-situ; *TD*, thyroid dysgenesis.

We specifically observed that, among the variants detected in patients, 65.5% (19/29) harbored two variants, whereas 20.7% (6/29) harbored three variants. Although multisite variants are common in patients with CH, we noticed that 87.5% (21/24) of our patients harbored variants in a single gene, whereas only two patients and one patient harbored variants in two and three genes, respectively. Based on the number of mutated genes, we classified the patients into two distinct groups. The single-gene group comprised 26 cases (18 cases of GIS, 4 cases of goiter, and 4 cases of TD) with variants in a single gene (**Figure 2A** and **Table 3**). More specifically, we noticed that, in the single-gene group, 20 cases had *DUOX2* variants, three cases had *DUOXA2* variants, and three cases had *TSHR* variants. The multi-gene group comprised three GIS cases, namely, two cases that harbored variants in *DUOX2* and *TSHR*, and *SLC26A4* and *TPO*, respectively, and one case that harbored variants in *DUOXA2*, *TPO*, and *TSHR* (**Table 3**).

**Table 3:** Clinical description and variants in patients with monoallelic, biallelic, and oligogenic variants.

Patient ID	Sex	Heel blood TSH (mIU/L)	TSH at diagnosis (mIU/L)	FT4 at diagnosis (pmol/L)	Initial dose (μg)	Dose at 1 year of age (μg)	Current dose (μg)	Thyroid morphology	Controllable reduction of L-T4 dose	Variants	Inheritance
2^a^	Female	79.1	49.6	2.86	33.5	12.5	12.5	Normal	Yes	DUOXA2, c.413dupA, p.Tyr138*	Father
13	Female	128	>75	5.1	50	25	62.5	Hypoplasia	No	TSHR, c.1574T>C, p.Phe525Ser	Father
18	Male	122	34.15	2.5	50	33.5	50	Normal	No	DUOX2, c.1087A>G, p.Ser363Gly	Father
21	Female	379	>75	2.6	37.5	50	50	Hypoplasia	No	DUOX2, c.2335G>A, p.Val779Met	Father
1^a^	Male	51	>75	0.99	50	37.5	50	Normal	No	DUOXA2, c.738C>G, p.Tyr246*	Mother
										DUOXA2, c.413dupA, p.Tyr138*	Father
3	Female	70.3	>75	4.88	50	12.5	25	Goiter	No	DUOX2, c.3329G>A, p.Arg1110Gln	Mother
										DUOX2, c.596del, p.Ser199Trpfs*122	Father
4	Female	58	12.06	6.54	40	12.5	12.5	Normal	Yes	DUOX2, c.1588A>T, p.Lys530*	Father and mother
										DUOX2, c.3526C>T, p.Pro1176Ser	Mother
5	Male	116	>75	1.29	50	40	40	Normal	No	DUOX2, c.1588A>T, p.Lys530*	Father
										DUOX2, c.3478_3480delCTG, p.Leu1160del	Mother
6	Female	38.3	>75	1.54	40	25	33.5	Normal	No	DUOX2, c.3329G>A, p.Arg1110Gln	Father and mother
7	Male	118	10	4.46	33.5	16.5	16.5	Normal	Yes	DUOX2, c.1304A>G, p.Asp435Gly	Mother
										DUOX2, c.2048G>T, p.Arg683Leu	Father
										DUOX2, c.2033A>G, p.His678Arg	Father
9	Male	24.7	49.6	4.7	40	20	20	Goiter	Yes	DUOX2, c.1588A>T, p.Lys530*	Father
										DUOX2, c.2048G>T, p.Arg683Leu	Mother
										DUOX2, c.4027C>T, p.Leu1343Phe	Mother
10	Male	12.8	>75	6.78	50	30	25	Normal	Yes	DUOX2, c.1606C>T, p.Arg536*	Mother
										DUOX2, c.1588A>T, p.Lys530*	Father
11	Female	57	>75	0.42	35	16.5	16.5	Normal	Yes	DUOX2, c.1588A>T, p.Lys530*	Mother
										DUOX2, c.2033A>G, p.His678Arg	Father
12	Male	18.4	49.6	5.28	35	12.5	12.5	Goiter	Yes	DUOX2, c.3329G>A, p.Arg1110Gln	Father
										DUOX2, c.959T>C, p.Leu320Pro	Mother
15	Male	11.9	66.73	6.07	50	16.5	10	Normal	Yes	DUOX2, c.4405G>A, p.Glu1469Lys	Mother
										DUOX2, c.3721A>T, p.Ile1241Phe	Father
16	Female	25.8	21.91	6.3	33.5	25	20	Hypoplasia	No	TSHR, c.823G>A, p.Ala275Thr	Father
										TSHR, c.1349G>A, p.Arg450His	Mother
17	Female	116	75	1.08	50	20	25	Normal	No	DUOX2, c.3632G>A, p.Arg1211His	Father
										DUOX2, c.3329G>A, p.Arg1110Gln	Mother
19	Male	93.1	49.6	1.33	40	30	30	Normal	Yes	DUOX2, c.2921G>A, p.Arg974His	Father
										DUOX2, c.1588A>T, p.Lys530*	Mother
22	Female	70	70	1.23	25	25	25	Normal	No	DUOX2, c.3778G>A, p.Asp1260Asn	Mother
										DUOX2, c.227C>T, p.Pro76Leu	Father
23	Male	126	>75	3.96	37.5	16.5	12.5	Normal	Yes	DUOX2, c.1588A>T, p.Lys530*	Father
										DUOX2, c.244C>A, p.Arg82Ser	Mother
24	Female	136	>75	7.2	30	25	33.5	Normal	No	DUOX2, c.1588A>T, p.Lys530*	Mother
										DUOX2, c.3329G>A, p.Arg1110Gln	Father
25	Female	133.2	>75	3.18	50	12.5	12.5	Goiter	Yes	DUOX2, c.3329G>A, p.Arg1110Gln	Father
										DUOX2, c.602dupC, p.Gln202Thrfs*99	Mother
26	Female	33.3	14.02	5.9	35	16.5	33.5	Ectopy	No	TSHR, c.1736C>T, p.Ala579Val^b^	Father
										TSHR, c.2066T>G, p.Val689Gly	Mother
27	Male	25.6	49.6	4.46	40	25	25	Normal	Yes	DUOX2, c.3329G>A, p.Arg1110Gln	Mother
										DUOX2, c.2654G>A, p.Arg885Gln	Father
28	Female	37.7	>75	3.89	40	25	20	Normal	Yes	DUOXA2, c.738C>G, p.Tyr246*	Mother
										DUOXA2, c.413dupA, p.Tyr138*	Father
29	Female	128	>75	2.39	50	16.5	15	Normal	Yes	DUOX2, c.3329G>A, p.Arg1110Gln	Mother
										DUOX2, c.2654G>A, p.Arg885Gln	Father
8	Female	130	>75	1.75	33.5	33.5	33.5	Normal	No	TPO, c.1477G>A, p.Gly493Ser	Mother
										TPO, c.1949G>A, p.Gly650Glu	Father
										SLC26A4, c.1286C>A, p.Ala429Glu	Mother
14	Male	24.7	73.99	2.7	25	40	40	Normal	No	DUOXA2, c.739G>C, p.Gly247Arg	Mother
										TSHR, c.1574T>C, p.Phe525Ser	Mother
										TPO, c.2735dupT, p.Gln913Profs	Mother
20	Male	46.1	53.78	1.89	50	40	50	Normal	No	DUOX2, c.2894C>T, p.Ser965Leu	Mother
										DUOX2, c.2654G>A, p.Arg885Gln	Father
										TSHR, c.1574T>C, p.Phe525Ser	Father

TSH, thyroid-stimulating hormone; FT4, free thyroxine; L-T4, levothyroxine.

a Patient 1 and patient 2 are siblings. Patients 2, 13, 18, and 21 were monoallelic variants, and patients 8, 14, and 20 were oligogenic variants. The remaining patients were biallelic variants.

b c.1736C>T in TSHR is novel.

*Stop-gain variant.

We subsequently analyzed the differences in the clinical data between the single-gene and multi-gene groups (**Table 4**). We did not observe any differences in thyroid morphology, heel blood TSH at screening, and TSH or FT4 at diagnosis (*p* > 0.05). We further collected data on the L-T4 dose, including the initial dose, dose at 1 years of age, and current dose. Interestingly, we found that the dose at 1 year of age was significantly higher in the multi-gene group than that in the single-gene group (*p* = 0.018). However, we did not detect a significant difference between the initial and current doses. In addition, we identified a controllable reduction in the L-T4 dose in 53.8% of the patients in the single-gene group; however, no patients with such reduction in the L-T4 dose were observed in the multi-gene group.

**Table 4:** Comparison of the clinical characteristics in the single-gene and multi-gene groups.

	Single-gene group	Multi-gene group	*p*-value
Heel blood TSH (mIU/L)	70.15 (34.40–121.00)	46.10 (35.40–88.05)	0.802
TSH at diagnosis (mIU/L)	>75.00 (49.60–75.00)	73.99 (63.88–74.50)	0.877
FT4 at diagnosis (pmol/L)	3.92 (1.75–5.23)	1.89 (1.82–2.29)	0.283
Initial dose (μg)	40.00 (35.00–50.00)	33.50 (29.25–41.75)	0.299
Dose at 1 year of age (μg)	22.50 (16.50–25.00)	40.00 (36.75–40.00)	0.018
Current dose (μg)	25.00 (15.38–33.50)	40.00 (36.75–45.00)	0.066

TSH, thyroid-stimulating hormone; FT4, free thyroxine.

### Characteristics in the Monoallelic, Biallelic, and Oligogenic Groups

Our aforementioned results suggested that multisite variants in a single gene prevailed in patients with CH. Therefore, we analyzed the pattern of the detected variants in their parents using Sanger sequencing. Based on the pattern of the detected variants, we classified the patients into three groups. Patients with variants in a single gene were subdivided into a monoallelic and a biallelic group. As shown in **Figure 2B**, the biallelic group comprised 22 cases with several variants in a single gene, including homozygous or compound heterozygous variants. The monoallelic group included four cases with a single variant in a single gene. In the biallelic group, we found that 81.8% (18/22) of cases (16 cases of GIS, 4 cases of goiter, and 2 cases of TD) harbored variants in *DUOX2*, two GIS cases had *DUOXA2* variants, and two TD cases had *TSHR* variants. In the monoallelic group, we detected two cases with *DUOX2*, one GIS case with *DUOXA2*, and one TD case with *TSHR*. Of note is that the components of the cases in the oligogenic group were the same as those in the multi-gene group (**Table 3**).

The levels of heel blood TSH at screening, TSH and FT4 at diagnosis, and the initial, 1 year, and current L-T4 doses in the biallelic, oligogenic, and monoallelic groups are shown in **Table 5**. Considering the small sample size of each group, we did not perform comparisons among the three groups. We observed a controllable reduction in the L-T4 dose in 25% (1/4) of patients in the monoallelic group and in 59.1% (13/22) of patients in the biallelic group; however, we did not observe such a reduction in patients in the oligogenic group.

**Table 5:** Comparison of the clinical characteristics in the monoallelic, biallelic, and oligogenic groups.

	Monoallelic group	Biallelic group	Oligogenic group
Heel blood TSH (mIU/L)	125.00 (111.28–190.75)	57.50 (27.68–116.00)	46.10 (35.40–88.05)
TSH at diagnosis (mIU/L)	62.30 (45.74–75.00)	75.00 (49.60–75.00)	73.99 (63.88–74.50)
FT4 at diagnosis (pmol/L)	2.73 (2.58–3.42)	4.21 (1.38–5.75)	1.89 (1.82–2.29)
Initial dose (μg)	43.75 (36.50–50.00)	40.00 (35.00–50.00)	33.50 (29.25–41.75)
Dose at 1 year of age (μg)	29.25 (21.88–37.62)	20.00 (16.50–25.00)	40.00 (36.75–40.00)
Current dose (μg)	50.00 (40.62–53.12)	22.50 (15.38–28.75)	40.00 (36.75–45.00)

TSH, thyroid-stimulating hormone; FT4, free thyroxine.

## Discussion

Our study demonstrated the existence of distinctive characteristics of genetic defects in different patients with CH. Historically, 75%–85% of CH cases have been attributed to TD, with the remainder occurring due to DH (10). However, patients with normal-sized GIS accounted for the majority of the cases in our cohort. Meanwhile, the frequency of genetic defects in the genes causing DH was higher than that in the genes causing TD, which was in agreement with previous studies in China and other Asian countries (11–13). These studies suggested that DH might be the leading pathophysiology of CH in Chinese populations, in contrast to what has been reported in other populations. We assumed that this difference might be a result of the genetic backgrounds of different ethnic populations.

In the present cohort, variants were most frequently identified in *DUOX2*, followed by *TSHR* and *DUOXA2*. The higher frequency of variants in *DUOX2* was consistent with the observation of a higher proportion of DH cases in our participants. Variants in *DUOX2* have been frequently reported, especially in East Asia (11–14). Several studies have suggested that the most reported variants among Chinese, Japanese, and Thai patients with CH have been identified in *DUOX2* (12, 13, 15), suggesting that *DUOX2* variants are an even more frequent causative factor for CH than previously recognized. In particular, the p.Lys530* and p.Arg1110Gln variants have been reported in Asian populations, including Chinese (16–18), Japanese (19, 20), Korean (21), and Malaysian (22) patients. The p.Lys530* and p.Arg1110Gln variants in *DUOX2* were also identified as the most frequent sites with a rate of 19% in this study population.

Although multisite variants are known to be common in patients with CH, in our cohort, most multisite variants were detected in a single gene, i.e., *DUOX2*. By detecting the respective variants in the parents of probands, we identified both monoallelic and biallelic *DUOX2* variants; the proportion of biallelic *DUOX2* variants was overwhelmingly higher. Monoallelic mutations are thought to confer phenotypes due to haploinsufficiency (23). Monoallelic *DUOX2* mutations have been reported to result in mild transient CH (16, 24, 25), whereas biallelic *DUOX2* mutations cause severe permanent CH. However, subsequent studies have reported biallelic mutations in patients with transient CH (19, 26) and monoallelic mutations in patients with permanent CH (17, 27). Moreover, patients with *DUOX2* mutations have been gradually recognized as exhibiting phenotypic heterogeneity (28, 29). In our previous study, we confirmed that the number of *DUOX2* variants could not be used to predict the transient or permanent outcomes (30). However, the correlation between variants and hormone characteristics or L-T4 dose remains unclear. Therefore, in the present study, the levels of hormones and the dose of L-T4 were used to analyze the correlation between variants and the phenotypic characteristics.

Application of next-generation sequencing (NGS) revealed that a significant proportion of patients harbored variants in more than a single gene (31–33). Based on these findings, the oligogenic model and the viewpoint of combinational mutant genes resulting in the pathogenesis of CH were proposed. In particular, oligogenicity implied that the pathogenesis of CH could be attributed to the sum of mutations. We detected multi-gene variants, namely, oligogenic variants, in 7.1% of patients in our cohort. Among these patients, patient 14 harbored oligogenic variants on one allele. Considering that the mother of patient 14 has the same variants showing symptoms of hypothyroidism, we kept patient 14 in the follow-up analysis. To date, data on the detection rates of oligogenic variants have been rarely reported. In a study in the Japanese population, oligogenic variants were identified in 18.0% of patients with CH using targeted NGS of 24 causative genes (15). Another study in the Italian population reported that oligogenic defects were found in 26.2% of the cohort using targeted NGS of 11 causative genes (34). Given that differences in ethnic populations have been shown to result in differences in the mutational spectrum, we speculated that the oligogenicity characteristics might be distinctive among different populations.

The oligogenic model has been suggested as the genetic etiology of CH, on the basis that it provides a suitable explanation for the complex forms of inheritance and the variable expressivity of mutations in CH (31, 35). More specifically, the coexistence of mutations in multiple genes might contribute to the severity of the hypothyroid condition and lead to great genotype–phenotype variability (15, 36). However, this hypothesis has not been fully clarified yet. To address this, we compared the clinical data between the single-gene and multi-gene groups. However, we did not observe any differences in the levels of heel blood TSH at NBS or those of serum TSH and FT4 at diagnosis. We also analyzed the differences in the L-T4 treatment doses at different stages (initial, 1 year of age, and current). Our results demonstrated that only the L-T4 treatment dose at 1 year of age was significantly higher in the multi-gene group. Furthermore, we divided the single-gene group into monoallelic and biallelic groups according to the number of variants. Considering the small sample size of each group, we did not perform statistical analysis among the three groups. In the treatment, the L-T4 doses of some patients could be easily controlled in order to keep the thyroid hormones in the normal range; however, this was not easy in some patients. We wondered whether it is related to the genotype. Regarding the change in the treatment dose, we noticed that fewer patients in the oligogenic group had a controllable reduction. However, it is still uncertain whether relatively high doses of L-T4 were administered to patients with oligogenic variants due to the limitation of the small sample size in this study. We will continue to focus on the study of CH.

Previous studies have suggested a detection rate of approximately 20% for CH candidate genes in Japanese and Czech cohorts with CH (15, 37). Using efficient sequencing methods, such as multiplex PCR and targeted NGS, recent studies have reported an increased detection rate of CH candidate genes (14, 16, 33), especially in patients with DH (38). However, the detection rate of variants in TD cases has been shown to be only approximately 5% (3, 39). Unlike TD, DH appears to have a detectable genetic basis in many cases (40, 41). Using WES, the variant detection rate was 69.0% in our cohort. This was consistent with the results of previous studies in the Chinese population using targeted NGS (ranging from 52% to 65%) (14, 33). Efficient sequencing methods and ethnic differences might have contributed to the high detection rates in Chinese cohorts (41). In addition, some ambiguous variants were confirmed as disease-causing variants. Advances in the study of CH candidate genes help to increase the detection rate of variants in later research.

Our results might help elucidate the mechanisms underlying the pathogenesis of CH. However, certain limitations were noted in our study. Firstly, the small sample size of the oligogenic and monoallelic groups might have limited the statistical performance. Secondly, we were unable to detect intronic variants and verify the function of the variants of uncertain significance. Therefore, further studies are required to clarify the molecular etiology and genotype–phenotype correlations in CH.

## Data Availability Statement

The original contributions presented in the study are included in the article/**Supplementary Material**. Further inquiries can be directed to the corresponding authors.

## Ethics Statement

The studies involving human participants were reviewed and approved by the ethics committee of Nanjing Medical University. Written informed consent to participate in this study was provided by the participants’ legal guardian/next of kin.

## Author Contributions

WL, FG, and YW participated in the diagnostic workup. HW, YW, and PX participated in the interpretation of clinical and biochemical data. RY, WL, and FG performed and interpreted the genetic analysis. WL and FG performed the statistical analysis.WL and BY wrote the manuscript. All authors contributed to the article and approved the submitted version.

## Funding

This work was supported by the National Natural Science Foundation of China (grant no. 81903400), Young Talent Plan of Changzhou Health Commission (grant no. CZQM2020096), Changzhou Key Laboratory of High-tech Research (grant no. CM20193009), and Changzhou Social Development Science and Technology Support Project (grant no. CE20205035).

## Conflict of Interest

The authors declare that the research was conducted in the absence of any commercial or financial relationships that could be construed as a potential conflict of interest.

## Publisher’s Note

All claims expressed in this article are solely those of the authors and do not necessarily represent those of their affiliated organizations, or those of the publisher, the editors and the reviewers. Any product that may be evaluated in this article, or claim that may be made by its manufacturer, is not guaranteed or endorsed by the publisher.
